# Epidémiologie et diagnostic de la lithiase urinaire: étude transversale dans une population camerounaise

**DOI:** 10.11604/pamj.2023.45.61.38677

**Published:** 2023-05-26

**Authors:** Landry Oriole Mbouché, Achille Aurèle Mbassi, Jean Crepin Eloundou Nkolo, Josepha Abe Avebe, Justin Kamga, Pierre Joseph Fouda, Fru Angwafo III

**Affiliations:** 1 Service de Chirurgie et Spécialités, Hôpital Gynéco-Obstétrique et Pédiatrique de Yaoundé, Yaoundé, Cameroun,; 2 Département de Chirurgie et Spécialités, Faculté de Médecine et des Sciences Biomédicales de l´Université de Yaoundé I, Yaoundé, Cameroun,; 3 Service d´Urologie, Hôpital Central de Yaoundé, Yaoundé, Cameroun,; 4 Département de Chirurgie, Institut Supérieur de Technologie Médicale de Yaoundé, Yaoundé, Cameroun,; 5 Service d´Urologie, Hôpital Militaire de Yaoundé, Yaoundé, Cameroun,; 6 Service de Chirurgie, Hôpital Général de Yaoundé, Yaoundé, Cameroun

**Keywords:** Lithiase urinaire, fréquence, diagnostic, Yaoundé, Urinary lithiasis, frequency, diagnosis, Yaoundé

## Abstract

**Introduction:**

la lithiase urinaire est une maladie multifactorielle qui se caractérise par la présence de concrétions dans les voies excrétrices. Le diagnostic de la lithiase urinaire repose sur la clinique et l´imagerie médicale. L´objectif de cette étude était de déterminer le profil épidémiologique et clinique des patients souffrant de lithiase dans la ville de Yaoundé.

**Méthodes:**

il s´agissait d´une étude observationnelle descriptive de type transversale avec une collecte de données rétrospective. Ont été inclus dans notre étude, les dossiers des patients avec un âge >15 ans, les patients diagnostiqués de lithiase confirmés par une imagerie médicale. À partir d´un questionnaire préalablement établi, les variables étudiées étaient sociodémographiques, cliniques et paracliniques.

**Résultats:**

au total 120 patients ont été inclus dans notre étude. L´âge moyen était de 40,46 ans ±12,62 ans avec des extrêmes de 19 à 74 ans. Le sexe masculin était prédominant à 60,8% (n=73). La colique néphrétique était la circonstance retrouvée dans 67,5% (n=81). L´examen physique était normal dans 55,8% (n=67). L´hydratation insuffisante était le facteur associé le plus retrouvé chez 45% (n=61). L´uroscanner a été l´examen le plus réalisé dans notre étude, soit 50,7% (n=61). La médiane de la densité des calculs était de 731 Unités Hounsfield [346; 1183,5]. Les calculs siégeaient majoritairement dans le haut appareil. La topographie rénale gauche était prépondérante dans 35% (n=42) des cas. L´examen cytobactériologique des urines retrouvait Escherichia Coli à 60% (n=15). Sur le plan métabolique, la calcémie, la phosphorémie et l´uricémie ont été demandées respectivement dans 15,8% (n=19), 0,8% (n=1) et 12,5% (n=15) des cas. Les résultats étaient normaux. En l´absence de laboratoire spécialisé dans l´analyse biochimique du calcul, une minorité des patients (n=3) a bénéficié d´une spectrophotométrie.

**Conclusion:**

la lithiase urinaire est une maladie de l´homme de la quarantaine. La colique néphrétique est le principal signe révélateur. Le diagnostic est révélé par le scanner abdominal dans la moitié des cas. Le bilan métabolique et l´analyse constitutionnelle du calcul restent très faiblement demandés.

## Abstract (en)

**Introduction:**

urinary stone is a multifactorial disease characterized by the presence of concretions in the urinary tract. The diagnosis of urinary lithiasis is based on clinical and imaging findings. The objective of this study was to determine the profile of patients suffering from urolithiasis in the city of Yaounde.

**Methods:**

this was a descriptive observational cross-sectional study with retrospective data collection. Patients with an age > 15 years and patients diagnosed with lithiasis confirmed by medical imaging were included in our study. The variables studied were sociodemographic, clinical and paraclinical.

**Results:**

a total of 120 patients were included in our study. The mean age was 40.46±12.62 years with extremes from 19 to 74 years. Male gender was predominant at 60.8% (n=73). Renal colic was the circumstance found in 67.5% (n=81). Physical examination was normal in 55.8% (n=67). Insufficient hydration was found in 45% (n=61) of cases. Abdominal scanner was the most performed examination in our study in 50.7% (n=61). The median stone density was 731 Hounsfield units [346; 1183.5]. The stones were predominantly located in the upper tract. The left renal topography was predominant in 35% (n=42) of cases. Urine culture revealed Escherichia Coli at 60% (n=15). Blood calcium, phosphorus and uricemia were requested in 15.8% (n=19), 0.8% (n=1) and 12.5% (n=15) of cases, respectively. The results were normal. In the absence of a laboratory specialized in biochemical analysis of calculi, a minority of patients (n=3) benefited from spectrophotometry.

**Conclusion:**

urinary lithiasis is a disease of men in their forties. Renal colic is the main revealing sign. The diagnosis is revealed by the abdominal scanner in half of the cases. Metabolic assessment and constitutional analysis of the calculus are still very rarely requested.

## Introduction

La lithiase urinaire est une maladie multifactorielle qui se caractérise par la présence de concrétions pierreuses dans les voies excrétrices. La population affectée est estimée à 2 à 3% dans le monde [1]. Le risque de développer une lithiase urinaire chez les adultes semble être plus élevé dans l'hémisphère occidental (5-9% en Europe, 12% au Canada, 13-15% aux États-Unis) que dans l'hémisphère oriental (1-5%), bien que les risques les plus élevés aient été signalés dans certains pays asiatiques comme l'Arabie Saoudite (20,1%) [2,3]. L'incidence de la lithiase urinaire dans une population donnée dépend de la zone géographique, de la répartition raciale et du statut socio-économique de la communauté. L'évolution des conditions socio-économiques au fil du temps, et les changements subséquents dans les habitudes alimentaires, ont affecté non seulement l'incidence, mais aussi le site et la composition chimique des calculs [4]. Dans les pays en voie de développement, notamment la ceinture lithiasique afro-asiatique qui regroupe plusieurs pays [Soudan, Egypte, Arabie Saoudite, Emirats Arabes Unis, Iran, Pakistan, Birmanie, Inde, Thaïlande, Indonésie, Philippines] la prévalence varie de 4 à 20% et touche une population âgée de plus de 70 ans avec un sexe ratio de 2 en faveur des hommes [4]. En Afrique, l´épidémiologie de la lithiase urinaire est peu connue [5] du fait du faible taux de fréquentation des structures de santé et l´absence d´étude étendue dans la population générale conduiraient à une sous-estimation de cette pathologie. Les manifestations cliniques ont une présentation polymorphe dépendant du siège et du degré d´obstruction de la lithiase. Le diagnostic repose sur l´imagerie médicale qui permet de mettre en évidence la topographie, le nombre et la taille des calculs, ainsi que son retentissement sur les voies excrétrices et le parenchyme rénal. Notre étude avait pour but d´étudier les aspects épidémiologiques, diagnostiques des lithiases urinaires dans cinq hôpitaux de la ville de Yaoundé.

## Méthodes

**Type d´étude:** nous avons mené une étude observationnelle descriptive, de type transversale, avec une collecte de données rétrospective.

**Lieu et période de l´étude:** l´étude a été réalisée au sein de cinq hôpitaux de première catégorie de la ville de Yaoundé, à savoir, Hôpital Gynéco-Obstétrique et Pédiatrique, Hôpital Général, Hôpital Militaire, Hôpital Central et Clinique Fouda. La durée de l´étude de 11 ans s´étendait du 1^er^ janvier 2011 au 31 mai 2022.

**Population d´étude:** étaient inclus, tous les dossiers des patients âgés de plus 15 ans avec lithiase urinaire, confirmée par l´imagerie médicale et traitée dans l´un des sites retenus. Les dossiers incomplets et les patients perdus de vue étaient exclus. Pour le calcul de la taille de l´échantillon, nous avons utilisé la formule de Cochrane. En considérant une prévalence hospitalière de 17,4% [6], on trouve la taille minimale de l´échantillon à 226 patients.

**Collection des données:** pour la collecte des données, nous nous sommes rendus aux archives des différents services de chirurgies et spécialités des hôpitaux, où a été menée notre étude, les patients remplissant nos critères furent sélectionnés. Pour les dossiers incomplets, nous contactions les patients par téléphone et parfois en présentiel. Nous avons recueilli les informations par l´intermédiaire d´une fiche technique préalablement établie.

**Variables:** les données collectées étaient sociodémographiques, notamment le sexe, l´âge, la profession, les habitudes alimentaires et les antécédents de lithiase urinaire dans la famille. Sur le plan clinique étaient recherchés le motif de consultation, les signes digestifs et urinaires, la pression artérielle, la température, état d´hydratation et les découvertes de l´examen physique. Les données paracliniques étaient constituées de la créatinine sérique, urée plasmatique, glycémie, numération formule sanguine, ionogramme sanguin, examen cytobactériologique des urines, le volume urinaire des 24 heures, le calcium, l´acide urique, le sodium, urée, pH urinaire, échographie abdominale, la radiographie de l´arbre urinaire sans préparation, scanner abdominal, urographie intraveineuse.

**Analyse des données:** nous avons utilisé Microsoft Word, Excel et les outils statistiques (logiciels SPSS version 23.0, CS Pro 7.6.0 et MS Excel 2010) pour l´analyse. Les variables quantitatives ont été décrites par la moyenne, l´écart type, la médiane et l´interquartile. Les variables qualitatives quant à elles ont été présentées sous forme de fréquence et pourcentage.

**Considérations éthiques:** la clairance éthique a été obtenue du Comité d´Éthique Institutionnel de la recherche pour la santé humaine de l´Université de Douala (N°3294 CEI-Udo/06/2022/T). Nous avons également obtenu les autorisations de recherche dans les différents hôpitaux.

## Résultats

Au cours de la période d´étude, 269 dossiers ont été inclus, parmi lesquels 149 ont été rejetés de l´étude pour dossiers incomplets (n=90 dossiers), numéros de téléphones indisponibles (n=30) et indisponibilité des patients (n=29). Au total, 120 dossiers ont été définitivement inclus dans notre étude.

**Figure 1:**
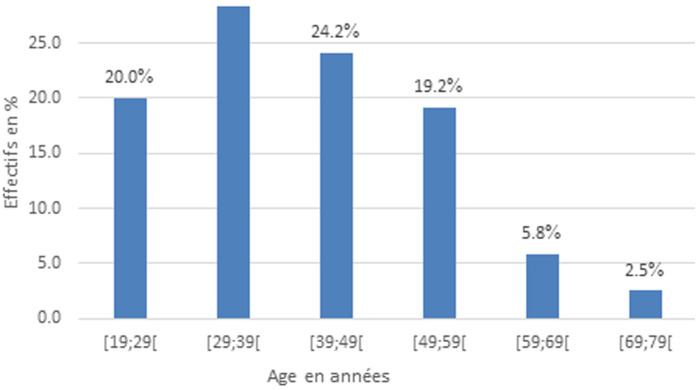
tranche d´âge

**Caractéristiques socio-démographiques:** la tranche d´âge la plus représentée variait de 29 à 39 ans, soit 28,3% (n=34) (Figure 1). L´âge moyen était de 40,46 ± 12,62 ans, avec des extrêmes allant de 19 à 74 ans. Les hommes étaient les plus affectés dans notre série, soit 60,8% (n=73). Les fonctionnaires étaient les plus représentés dans la profession, soit 25,8% (n=31).

**Figure 2:**
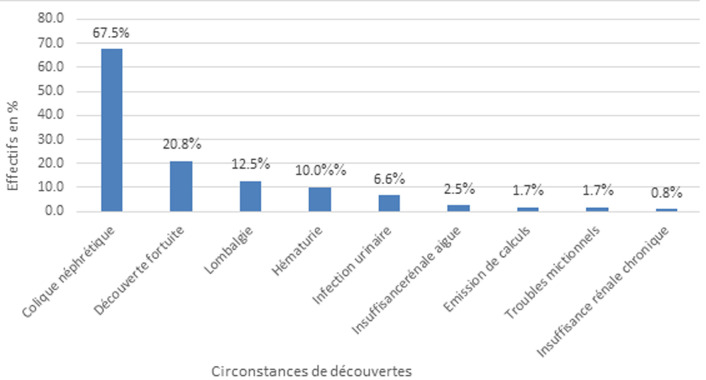
circonstances de découverte

**Figure 3:**
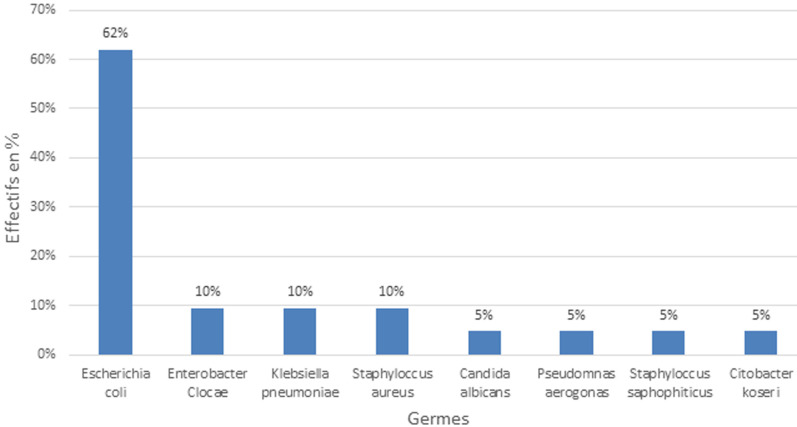
germes identifiés à la culture des urines

**Caractéristiques cliniques et paracliniques:** la colique néphrétique était la circonstance de découverte la plus retrouvée dans 67,5% (n=81) des cas (Figure 2). L´hydratation insuffisante qui représentait 45% (n=61) était le facteur associé prépondérant. Les facteurs associés à la lithiase urinaire se trouvent dans le Tableau 1. A l´examen physique, la sensibilité des flancs était le signe physique le plus retrouvé dans 95,3% (n=114) des cas. L´examen physique était normal chez 55,8% (n=67) patients. L´uro-scanner était l´examen complémentaire morphologique le plus réalisé dans les différents hôpitaux dans 50,7% (n=61) des cas. Concernant la topographie des calculs, le rein gauche était plus atteint par rapport au côté droit dans 35% (n=42) des cas. Le Tableau 2 résume la localisation des calculs dans les voies urinaires. Au niveau de l´uretère, l´uretère pelvien droit, soit 6% (n=15) était la partie la plus atteinte. La vessie représentait 11% (n=13) des localisations. Le nombre moyen de calculs par patient était de 2±1,83 avec des extrémités allant de 1 à 8. La taille médiane des calculs était de 9 mm [5-15]. La médiane de la densité des calculs était 731 Unités Hounsfield [346;1183,5]. L´examen cytobactériologique des urines était positif chez 25 patients (20,8%), le germe le plus fréquemment retrouvé a été *Escherichia Coli* chez 60% des patients (Figure 3). Sur le plan métabolique, la calcémie, la phosphorémie et l´uricémie ont été demandées respectivement dans 15,8% (n=19), 0,8% (n=1) et 12,5% (n=15) des cas. Les résultats étaient normaux. En l´absence de laboratoire spécialisé dans l´analyse biochimique des calculs urinaires, une minorité de nos patients (n=3) a bénéficié d´une spectrophotométrie. Le pH urinaire moyen était de 5,8±0,8 avec un minimum de 5 de 7.

**Tableau 1:** facteurs associés à la lithiase urinaire

Facteurs associés	Effectifs (n=136)	Pourcentage
Hydratation insuffisante	61	45%
Immobilisation prolongée	18	13%
Régime carné	12	9%
Thé	11	8%
Voyage récent et prolongé	10	7%
Produits Laitiers	8	6%
Régime salé	6	4%
Café	5	4%
Lait	5	4%

**Tableau 2:** topographie du calcul

Topographie		Effectifs (n = 209)	Pourcentage
Reins	Gauche	74	35%
	Droit	71	34%
	Bilatéral	10	5%
Uretére pelvien	Droit	15	6%
	Gauche	4	2%
Uretére lombaire	Droit	3	1%
	Gauche	2	1%
Uretére iliaque	Droit	9	4%
	Gauche	9	4%
Jonction urétéro vésicale		1	1%
Vessie		11	5%

## Discussion

L´objectif général de notre étude était de mettre en avant le profil épidémiologique, diagnostique des lithiases urinaires dans cinq hôpitaux de la ville de Yaoundé. Nous avons enregistré 269 cas de lithiases urinaires. Cent vingt (120) cas répondant à nos critères d´inclusion furent définitivement sélectionnés, sur une durée d´étude allant du 1^er^ janvier 2011 au 31 mai 2022. La fréquence retrouvée dans notre étude était de 17,25%. Sur le plan clinique, la colique néphrétique était la circonstance de découverte la plus représentée, soit 67,5% (n=81). L´hydratation insuffisante était le facteur favorisant de lithiase dans 45% des cas. L´uroscanner était l´examen le plus réalisé, soit 50,7%. En France, Daudon *et al*. en 2008 ont rapporté une prévalence de 10% [7]. Diallo *et al*. au Sénégal à Dakar en 2015 ont retrouvé une prévalence de 18,42% pour les lithiases du haut appareil urinaire [8]. A Maroua au Cameroun, Ngaroua *et al*. avaient trouvé une prévalence de 17, 39% [6]. L´incidence de la lithiase urinaire variant d´un pays à un autre, Il est difficile de la retrouver avec précision, car de nombreux facteurs modifient les données recueillies [Habitudes alimentaires, situations géographiques, consanguinité…] [4]. L´âge moyen de nos patients était de 40,46±12,62. Des études faites dans trois pays notamment, aux Etats Unis en 2017 [9], au Congo 2022 [10] et au Cameroun en 2021 [11] rapportaient les mêmes résultats. Respectivement 40,4 ans, 42,05 ans et 40,95 ans. La lithiase urinaire est une pathologie qui apparait au cours de la période d´activité de travail chez les adultes. Cette fréquence serait élevée au cours cette période de vie, probablement en rapport avec le type de travail et les changements de mode de vie [12].

Le sexe masculin prédominait dans notre étude avec 60,8% (n=73). Notre résultat se rapproche de celui de Daudon *et al*. en France en 2008 [7], et Ngaroua *et al*. au Cameroun en 2017 [6]. La répartition des calculs selon le sexe est variable d´une étude à l´autre. C´est ainsi que Diagana en Mauritanie dans une série de 164 cas a trouvé un sexe ratio de 1/10 en faveur des femmes [13]. De l´autre côté, Laziri au Maroc rapportait un sexe ratio de 1/7 pour les hommes [14]. Concernant les manifestations cliniques, Abago *et al*. au Togo en 2021, retrouvaient dans 91,9% la colique néphrétique comme principale signe fonctionnel [15]. La topographie du calcul dans le haut appareil urinaire en est la principale explication car la majorité des calculs se forme dans les reins et une partie d´entre eux peut ensuite migrer dans l´uretère avant d´être expulsée par les voies naturelles [7]. Les modifications alimentaires constituent un risque important ou facteurs associé à la formation des calculs urinaires. Diangienda *et al*. à Kinshasa ont trouvé l´hydratation insuffisante à 68,2% [16]. Coulibaly *et al*. au Mali trouvaient que les produits laitiers étaient le facteur associé le plus représenté, soit 40,7% [17]. Selon les régions, les habitudes alimentaires varient et les facteurs favorisants de lithiases urinaires suivent les mêmes variations. Au Burkina Faso, Kaboré *et al*. ont rapporté un examen physique normal à 91,6% [5] Les signes cliniques généralement dus à un obstacle sur les voies urinaires dépendent de la topographie, du nombre, de la taille du calcul et de son caractère obstructif ou non. L´échographie de l´appareil urinaire était prescrite en première intention. Ce résultat s´oppose à celui de Niang *et al*. au Sénégal à Dakar en 2015 qui avaient rapporté une réalisation de l´uroscanner chez 95,6% des malades [18]. Cette étude concernait la fragmentation au Laser qui ne saurait être réalisée sans une imagerie précise. La prescription des examens varie d´un hôpital à l´autre. La précarité de l´économie des patients peut être un frein pour la réalisation de certains examens qui sont parfois onéreux. Cependant, le scanner abdominal sans injection de produit de contraste reste déterminant pour le diagnostic et le retentissement de la lithiase sur l´appareil urinaire. Les calculs urinaires se forment le plus souvent dans les reins [19].

Notre étude retrouvait une localisation rénale dans 71%. Par contre, Ngaroua *et al*. au Cameroun rapportaient une prévalence de 37% de lithiase rénale et une localisation prédominante de calcul dans la vessie (56,52%) [6]. Le côté rénal gauche était le plus atteint chez 35% de la population d´étude. C´est le cas de Muhindo *et al*. à Cotonou au Benin en 2021 (26,5%) et Djelloul *et al*. en Algérie en 2006 qui avaient rapporté respectivement 26,5%, 47,3% [20,21]. La prédominance du côté gauche ou droit dans la lithiase urinaire n´existe pas vraiment selon Daudon *et al*. [7]. Le nombre moyen du calcul dans notre étude était de 2± 1,83, Diamand *et al*. à Bruxelles ont retrouvé un résultat voisin du nôtre, soit 3,42 [22]. La taille médiane des lithiases dans notre étude était de 9mm [5; 15]. Ce résultat est comparable à celui de Coulibay *et al*. qui ont rapporté une variation de 6 à 10 mm [17]. Ces éléments nous renseignent sur la possibilité d´élimination spontanée du calcul. La médiane de la densité des calculs était, 731 [346; 1183,5] unités Hounsfield Bosquet *et al*. en France ont trouvé une densité moyenne de 713 UH [23]. La densité Hounsfield peut nous informer sur la composition chimique prédominante du calcul. En l´absence d´analyse chimique qui représente la clé du diagnostic de certitude de la composition du calcul, la lithiase urinaire aurait une prédominance calcique dans nos contrées selon cette densité retrouvée. L´examen cytobactériologique des urines était positif chez 25 patients dans notre série, le germe le plus retrouvé était *Escherichia Coli* soit 60%. Kuntima *et al*. en [24] et Kaboré *et al*. [5] avaient trouvé le même germe à 57,3% et 30% respectivement. Le pH urinaire moyen dans notre population était de 5,8±0,8. Ce résultat concorde avec celui de Pal *et al*. en 2021 à Pradesh en Inde qui retrouvaient un pH urinaire moyen de 6,6 [25]. La valeur du pH est importante dans la prise charge médicamenteuse des lithiases urinaires. Ainsi, en cas de pH acide, une alcalinisation des urines est indiquée. Nous avons réalisé une étude observationnelle descriptive, de type transversale, avec une collecte des données rétrospective dans cinq hôpitaux de la ville de Yaoundé. Les limites de cette étude sont dominées par le nombre élevé de dossiers inexploitables, l´absence de facteurs de risque lithiasique clairement établi et le défaut d´analyse morphologique et constitutionnelle des calculs.

## Conclusion

La fréquence de la lithiase urinaire était de 17,25% dans les cinq hôpitaux de Yaoundé. Les habitudes alimentaires sont dominées principalement par une hydratation insuffisante, principal facteur associé dans la formation de la lithiase urinaire dans notre étude. La colique néphrétique est le principal signe révélateur. Le diagnostic est révélé par le scanner abdominal dans la moitié des cas. Le bilan métabolique et l´analyse constitutionnelle du calcul restent très faiblement demandés.

### Etat des connaissances sur le sujet

- *L´incidence de la lithiase urinaire est en fréquente augmentation dans le monde;*
- *Les modifications alimentaires jouent un rôle favorable dans la formation des calculs;*
- *Le scanner abdominal est l´examen de référence dans le diagnostic et la composition du calcul est faite par l´analyse spectrophotométrique à l´infrarouge*.

### Contribution de notre étude à la connaissance

- *La lithiase du haut appareil urinaire est prépondérante à Yaoundé;*
- *L´hydratation insuffisante est l´un des facteurs favorisants;*
- *Les moyens de diagnostic moderne sont insuffisamment utilisés*.
